# 新型冠状病毒疫苗诱导的免疫性血栓性血小板减少症

**DOI:** 10.3760/cma.j.issn.0253-2727.2021.07.016

**Published:** 2021-07

**Authors:** 玉梅 刘, 化泉 王, 宗鸿 邵

**Affiliations:** 天津医科大学总医院血液科，天津 300052 Department of Hematology, General Hospital of Tianjin Medical University, Tianjin 300052, China

自新型冠状病毒肺炎（COVID-19）大流行开始至2021年5月末，全球记录在案的确诊病例数已超过1.7亿例，死亡病例数超过350万人，我国累计确诊病例超过11万例，死亡4900余人，经济和社会活动受到严重干扰。新型冠状病毒（SARS-CoV-2）疫苗是抗击COVID-19大流行最重要的应对措施。在一年内，已经开发出了几种疫苗，包括病毒载体疫苗、核酸疫苗、蛋白重组疫苗、灭活疫苗，并交付了数亿剂。我国上市的疫苗包括三种灭活疫苗和一种腺病毒载体疫苗。自2021年1月疫苗开始大范围接种至今，全球疫情控制得到了明显改善。而不良事件的报告是疫苗上市后的关键步骤。多个国家报告了接种阿斯利康SARS-CoV-2 ChAdOx1疫苗Vaxzevria及杨森/强生SARS-CoV-2 Ad26. COV2.S疫苗后出现的一种罕见但严重的血栓伴血小板减少事件，引起了广泛关注[1]–[2]。

这种血栓并发症可表现为严重的脑静脉窦血栓（CVST）、内脏静脉血栓、肺栓塞、动脉血栓形成等多发血栓事件，伴血小板减少、D二聚体升高、纤维蛋白原下降或正常，肝素/血小板因子4（PF4）抗体阳性。本病进展快，患者死亡率高达20％～50％[3]–[4]，需要引起高度重视。其发病机制与肝素诱导的血小板减少症（HIT）相似，称为疫苗诱导的免疫性血栓性血小板减少症（vaccine-induced immune thrombotic thrombocytopenia, VITT）[5]，美国食品和药物管理局（FDA）和疾病控制和预防中心（CDC）称之为“血栓伴血小板减少综合征（thrombosis with thrombocytopenia syndrome, TTS）。本文对此并发症进行概述，旨在提高医务人员及广大群众对此疾病的认识，并提高警惕。

一、流行病学

截至2021年4月4日，欧洲药物警戒数据库报告了在欧洲经济区（EEA）和英国接受第一剂阿斯利康ChAdOx1疫苗Vaxzevria的3400万人群中发生169例CVST和53例内脏静脉血栓病例，总体发病率约为6.5/10万。美国疫苗不良事件报告系统（VAERS）报告，18～49岁女性接种杨森/强生Ad26.COV2.S疫苗后血栓伴血小板减少事件的发生率为7/10万，50岁以上女性接种者的发生率为0.9/10万，在按年龄划分的亚组分析中（18～29、30～39、40～49、50～64、≥65岁），30～39岁组女性发病率最高（11.8/10万）[3]。而在未接种任何疫苗的一般人群中，静脉血栓的年发病率为1～2/1000，脑静脉血栓的年发病率为（0.22～1.57）/10万。因此，VITT的发病率并不高。但是，CVST合并VITT进展迅速，死亡率远远高于普通CVST（为5％～10％）[4],[6]–[7]。

VITT发病人群以18～49岁中青年女性为主，中位年龄约36岁，男性患者及50岁以上女性患者比例较小，这在两种疫苗中相似[4]。对VITT患者的潜在危险因素（包括肥胖、甲状腺功能减退、口服避孕药、高血压）进行分析，患者既往均无血栓性疾病、凝血功能异常病史，无血栓家族史，且发病前均未接触过肝素[3]。

二、发病机制

VITT的发病机制和致病因素尚不明确，但肝素/PF4抗体可能参与血栓形成。

根据欧洲和美国对阿斯利康Vaxzevria疫苗和杨森/强生SARS-CoV-2疫苗接种后报告的VITT患者进行的研究得出，在几乎所有患者血清中均检测出高水平的PF4抗体[4]。因此，VITT的发病机制可能与PF4抗体有关。

PF4抗体也称为肝素/PF4抗体，可在一小部分接触肝素的人中诱发血栓性血小板减少，称为肝素诱导的血小板减少症（HIT）。这提示VITT类似于严重的HIT。HIT是一种众所周知的血栓性疾病，由血小板活化抗体识别阳离子PF4和阴离子肝素形成的多分子复合物引起。然而，与经典的HIT不同，VITT患者发病前未接触过肝素。除肝素外的其他触发因子也可以引起血栓形成，在临床和血清学表现上类似于HIT，包括某些多聚阴离子药物（如硫酸戊聚糖、抗血管生成药物PI-88、高硫酸化硫酸软骨素）[8]。病毒和细菌感染[9]、手术后[10]–[11]也可发生HIT样自身免疫性血栓伴血小板减少。PF4-多聚阴离子抗体较常见，5％～7％的献血者可检测到PF4抗体[12]。但CVST或内脏静脉血栓形成较罕见。这表明我们对VITT发病机制的理解尚不完整，PF4抗体也可能在疫苗接种之前存在，因此二者之间的因果关系尚未明确，也不能确定是否应在所有疫苗接种者中检测PF4抗体。在今后的研究中应进一步在体内模型中直接证明PF4抗体可导致血栓和血小板减少。

在一些未接触肝素的COVID-19重症患者中也检测到了PF4抗体[13]，因此，在SARS-CoV-2众多的人同源序列中，可能有某一序列可以触发PF4抗体的产生。分子模拟可能在PF4抗体的产生中起关键作用。建议分析SARS-CoV-2刺突蛋白和PF4的一级序列及空间结构，以寻找潜在的同源性[14]。

这种PF4抗体介导的血小板激活能力可被高浓度肝素中和，也可被血小板膜Fcγ受体ⅡA（FcγRⅡA）（CD32a）的单克隆抗体阻断，该受体可诱导信号传递和血小板聚集/分泌、血小板-中性粒细胞和单核细胞复合体形成以及凝血酶生成。高浓度的多克隆免疫球蛋白也可中和这些自身反应性PF4抗体的血小板活化作用，从而证实血小板通过CD32a发挥免疫性过度活化作用[15]。

阿斯利康的黑猩猩ChAdOx1疫苗及杨森/强生的人Ad26.COV2.S疫苗都含有不具备复制能力的腺病毒载体，编码SARS-CoV-2刺突糖蛋白抗原。俄罗斯莫斯科Gamaleya研究所的Sputnik V疫苗和中国康希诺生物制品公司的Convidecia疫苗也是腺病毒载体疫苗。Sputnik V使用两种不同的血清型腺病毒Ad26和Ad5作为载体，间隔21 d，以克服人群中预先存在的腺病毒免疫力。Sputnik V疫苗Ⅲ期临床试验（纳入16 427例受试者）的中期分析显示，1例患者出现深静脉血栓形成，1例出现脑循环衰竭，1例出现短暂性脑缺血发作，1例出现脑血管病[16]。在撰写本文时，有关不良影响的细节尚未发表。而Convidecia疫苗尚无血栓事件报道，无相关文章发表。腺病毒可结合血小板并引起血小板活化，腺病毒载体也可触发PF4的释放，导致多细胞活化，大量生成凝血酶，血小板消耗和血栓形成[17]–[18]。但是注射0.5 ml腺病毒疫苗1～3周后似乎不太可能对患者的血小板活化产生影响。因此，腺病毒载体因素尚存争议。

SARS-CoV-2感染患者（尤其是重症患者）常处于高凝状态[19]–[20]，患者多存在D-二聚体升高，肺部病理常见血管内透明血栓形成。研究显示，血栓在ICU患者中的发生率高达31％[21]。重症患者约4.5％合并脑梗死。SARS-CoV-2感染后免疫失衡介导的细胞因子释放综合征（CRS）可导致弥漫性血管内凝血（DIC）[22]，机制可能涉及免疫系统过度活化、全身炎症反应失控、大量细胞因子（IL-1β、IL-6、IL-2、IL-2R、IL-7、IL-10、IL-12、IL-18、IL-33、IFN-α、IFN-γ、GSCF、TNF-α、TGF-β）和趋化因子（CXCL10、CXCL8、CXCL9、CCL2、CCL3、CCL5、IP-10、MCP1、MIP1A）产生并释放[23]–[24]，引起全身血管内皮损伤并激活促凝通路而导致血栓形成[25]。疫苗接种后的炎症反应也可能通过类似机制增加内皮细胞黏附性和组织因子的释放，触发凝血途径，导致血栓形成[9]。

三、临床表现及实验室检查

（一）发病时间

从接种疫苗到出现VITT相关症状的间隔为4～30 d。在杨森/强生Ad26.COV2.S疫苗的Ⅲ期研究中，44％的60岁以下患者在接种疫苗后第1周发生头痛，大部分患者的症状在2 d内出现，并在随后的2 d内消失[6]。若接种4 d以后出现症状应予以重视，尤其是症状持续者。

（二）临床表现

VITT患者主要表现为一处或多处不寻常部位血栓形成，包括CVST、内脏静脉血栓形成、动脉性血栓栓塞和血栓性微血管病（TMA）[2]。症状包括严重的头痛、视觉改变、持续腹痛、恶心呕吐、背部疼痛、胸痛、呼吸急促、腿部疼痛或肿胀、瘀点、容易擦伤或出血，如果在接种疫苗后的时间窗内出现以上情况，需要进行紧急医学评估。

患者还可能发生DIC、弥漫性出血症状，CVST患者可合并颅内出血，进展迅速，常为致命性的[6]。

（三）辅助检查

影像学检查提示血栓形成，可合并出血，重点检查CVST、内脏静脉血栓、肺栓塞和下肢静脉血栓。

血常规和外周血涂片提示血小板减少，平均血小板计数（20～40）×10^9^/L，范围为（10～110）×10^9^/L。大部分患者血浆D-二聚体升高，部分患者纤维蛋白原下降，国际标准化比值（INR）和活化部分凝血活酶时间（APTT）多处于正常范围，C反应蛋白水平多显著升高。

PF4抗体的酶联免疫吸附试验（ELISA）在几乎所有报告的患者中均呈高水平阳性。PF4抗体ELISA的阳性程度是否与VITT风险相关尚不清楚。添加或不添加PF4的患者血清均可诱导正常血小板的活化，添加PF4可增强血小板活化水平。Greinacher等[4]研究发现，添加低水平肝素（0.2 U/ml）并未增强血小板活化水平，反而血小板活化水平有下降趋势，这是VITT不同于经典HIT之处[4]，Scully等[26]的研究也得到一致结论。高浓度肝素（100 IU/ml）及静脉注射免疫球蛋白（IVIG）、血小板膜FcγRⅡA受体的单克隆抗体可阻断PF4抗体诱导的血小板活化。非ELISA法快速免疫试验（RIA）对VITT特异性欠佳，不建议使用。

经典HIT的功能分析试验可能有助于VITT的诊断，包括血清素释放试验（SRA）、肝素诱导的血小板活化试验（HIPA）、p-选择素释放试验等[27]。然而，美国研究者See等[6]对9例ELISA法检测PF4抗体阳性的VITT患者进行了HIT功能分析试验，仅1例患者SRA和p-选择素释放实验阳性。因此，这些验证实验在VITT中的实用性并不确切，结果不能排除假阴性。所有样本均应在任何治疗（如IVIG）前取样并保存，以便将来进行改进的验证实验。

报告的部分患者检测了抗磷脂抗体，多为阴性，少部分患者抗心磷脂抗体和抗β_2_糖蛋白1轻微升高[6]，狼疮抗凝物试验阴性，补体蛋白（C1q、C4、C3）水平和激活产物sC5b-9在正常范围内。抗凝血酶、蛋白C、蛋白S筛查结果阴性，ADAMTS13活性在受检患者中是正常的[28]。有些患者可表现为微血管病性溶血，但这不是患者的共同特征。

聚合酶链反应（PCR）检测患者的SARS-CoV-2核酸结果均为阴性，Scully等[26]对10例可获得的样本进行的SARS-CoV-2核衣壳蛋白抗体血清学检测结果为阴性，排除了近期SARS-CoV-2感染的证据。10例患者的刺突糖蛋白和受体结合域（RBD）的抗体水平均在一剂SARS-CoV-2疫苗接种者范围内，季节性冠状病毒抗体水平也在一剂SARS-CoV-2疫苗接种者和普通人群的范围内。

四、诊断

美国血液学年会（ASH）和CDC专家建议诊断VITT必须满足以下四条标准：发病时间为SARS-CoV-2疫苗（仅限杨森/强生、阿斯利康）接种后4～30 d，静脉或动脉血栓（通常为脑或腹部），血小板减少，PF4抗体（ELISA法）阳性。PF4抗体（ELISA）阴性可排除VITT。血小板减少不伴血栓形成且PF4抗体（ELISA）阴性，可能为免疫性血小板减少症（ITP）。

抗磷脂抗体、狼疮抗凝物试验、抗凝血酶筛查、ADAMTS13活性可协助除外其他血栓性疾病。SARS-CoV-2核酸PCR检测及核衣壳蛋白抗体检测可协助除外SARS-CoV-2感染。

五、治疗

如果确诊VITT，应转至三级医院诊治，并及时评估疾病进展程度。治疗药物包括以下几方面。

（一）非肝素抗凝治疗

虽然尚无研究证明肝素可加重血栓形成，但血液学家强烈建议VITT患者避免应用肝素抗凝，除非HIT试验为阴性。建议应用非肝素抗凝剂替代，直接凝血酶抑制剂（比伐鲁定、阿加曲班），磺达肝癸钠，阿哌沙班或利伐沙班可用于VITT患者的抗凝治疗[4]。对于血栓形成合并明显的凝血因子消耗的患者，抗凝治疗需要权衡利弊，尤其是在CVST患者中，出血可能是致命的，但不抗凝治疗可能同样有害。血栓患者应该接受至少三个月的抗凝治疗，与常规静脉血栓栓塞治疗一样。

（二）大剂量IVIG

应尽早给予大剂量IVIG（1 g·kg^−1^·d^−1^×2 d）。大剂量IVIG可抑制FcγR介导的血小板活化[4]。当患者出现严重的血小板减少和血栓形成时，大剂量IVIG可快速提高血小板计数，增加抗凝治疗的安全性。严重出血或需要手术的患者，可紧急给予IVIG。IVIG对SARS-CoV-2疫苗免疫应答的影响尚不完全清楚。但COVID-19大流行期间采集的血浆制备的IVIG中，可能存在SARS-CoV-2疫苗抗原的中和抗体而降低疫苗效力[29]，而且这些中和抗体会干扰SARS-CoV-2抗体的检测[29]。因此，建议使用COVID-19大流行前血浆制备的IVIG，避免疫苗失效。如果无IVIG，可考虑血浆置换。

（三）支持治疗

血小板输注可增加HIT患者死亡率[9]，基于与HIT的相似性，VITT患者应避免血小板输注。出血或低纤维蛋白原血症患者，可予纤维蛋白原替代治疗，纤维蛋白原应提高至1.5 g/L。

（四）靶向治疗

布鲁顿酪氨酸激酶（BTK）抑制剂（如伊布替尼），是批准用于B细胞淋巴瘤的药物，可多靶点作用于FcγRIIA激活的下游多个通路，已有研究证明BTK抑制剂可有效抑制FcγRIIA交联介导的血小板聚集，致密颗粒分泌、p-选择素表达和血小板-中性粒细胞聚集，被认为是VITT的另一个潜在治疗选择[30]。

（五）其他治疗

避免使用阿司匹林作为VITT的治疗或预防药物，阿司匹林在防止PF4抗体对血小板的激活方面效果不佳，且可能增加VITT患者出血的风险。已有一些病例中，同时使用糖皮质激素和IVIG，目前还没有共识或证据表明需要糖皮质激素治疗。

如果在疫苗接种后的时间窗内出现不寻常部位血栓形成伴血小板减少，不要等待PF4抗体的ELISA检测结果，而是立即给予非肝素抗凝剂和大剂量IVIG治疗[4]。

六、建议

英国药品和保健产品管理局（MHRA）、欧洲药品管理局（EMA）以及美国免疫实践咨询委员会（ACIP）分别对两种腺病毒载体疫苗进行了审查，两个机构都证实了与疫苗相关的血栓栓塞的风险并不高于普通人群的背景风险，并强调了疫苗对SARS-CoV-2非常有利的风险效益比，建议继续应用这两种疫苗[3],[26]。SARS-CoV-2疫苗接种的好处也在世界范围内得到认可，特别是在避免严重病例、显著限制住院人数、减少病毒传播和提供群体免疫方面[3]。

尽管如此，医务人员及广大群众应对疫苗不良反应（尤其是严重VITT）保持足够的警惕。应向疫苗接种者和广大群众提供SARS-CoV-2疫苗不良事件的教育，尤其是VITT的风险以及其他种类SARS-CoV-2疫苗的可选择性，特别是对18～49岁女性。在美国CVST病例报告中，从症状发作到住院的中位时间为7 d，提高患者和临床医生对VITT的认识可能会缩短进行临床评估和治疗的时间。临床医生如遇SARS-CoV-2疫苗后可疑的血栓伴血小板减少事件，考虑与疫苗相关后，应及时向药物警戒部门上报。疫苗接种者若在接种超过4 d后出现新发和持续的相关症状，应尽早就诊，以便进行及时并彻底的检查。

七、结语

SARS-CoV-2疫苗对于控制COVID-19大流行起到了关键作用，但疫苗相关严重不良反应需引起警惕。VITT虽罕见但进展迅速、死亡率高，需要引起广泛重视。其发病机制及如何监测尚需进一步研究。
